# PSMD3-ILF3 signaling cascade drives lung cancer cell proliferation and migration

**DOI:** 10.1186/s13062-023-00389-3

**Published:** 2023-06-19

**Authors:** Jin Zhang, Qianli Ma, Qiduo Yu, Fei Xiao, Zhenrong Zhang, Hongxiang Feng, Chaoyang Liang

**Affiliations:** Department of Thoracic Surgery, China-Japan Friendship Hospital, Number 2, Yinghua East Street, Chaoyang District, Beijing, 100029 China

**Keywords:** PSMD3, ILF3, Migration, Invasion, Ubiquitination

## Abstract

**Background:**

Proteasome 26S subunit, non-ATPase 3 (PSMD3) has been reported to participate in various human cancers. Nevertheless, the function of PSMD3 in lung cancer (LC) remains unclear.

**Methods:**

RT-qPCR and western blot were used to detect the expression of PSMD3 in LC tissues form TCGA database and clinical samples, and LC cell lines. To study the effect of PSMD3 on LC cell proliferation, migration, invasion, and apoptosis, siRNAs targeting PSMD3 were synthesized and overexpressed plasmids were constructed. CCK-8 assay, Transwell assay, and etc. were used to evaluate the results. Tumor xenograft model was used to evaluate the function of PSMD3 on tumor growth. CO-IP and MS were used to scan the proteins that bind with PSMD3. The interaction between PSMD3 and ILF3 in lung cancer cells were studied using IF staining, CHX protein stability, and ubiquitination assay. Additionally, the effect of ILF3 on cell progression and LC tumor growth was demonstrated by conducting a recovery assay using siILF3 and an ILF3 inhibitor YM155.

**Results:**

We observed that PSMD3 was significantly overexpressed in LC tissues and cells, which indicated a poor prognosis. Meanwhile, we found that PSMD3 promoted cell proliferation, migration, and invasion of LC cells. We also determined that PSMD3 stabilized the protein expression of ILF3 and the deubiquitination of ILF3 in lung cancer cells. Furthermore, animal experiments showed that the ILF3 inhibitor YM155 could suppress tumor growth with the presence of PSMD3.

**Conclusions:**

PSMD3 collectively regulated the stability of ILF3 protein and facilitated the ubiquitination of endogenous ILF3 in LC, which ultimately promoted the progression of LC cells. The PSMD3/ ILF3 axis could potentially be used as a novel strategy for both diagnosis and treatment of LC.

**Supplementary Information:**

The online version contains supplementary material available at 10.1186/s13062-023-00389-3.

## Background

Lung cancer (LC) is a prevalent malignant tumor that causes significant morbidity and mortality in China and worldwide [1, 2]. The five-year survival rate of LC is less than 20%, with tobacco smoking being a well-known risk factor for its development [3]. Non-tobacco-related risk factors include exposure to environmental and vocational exposures, lung infections, chronic lung disease, and lifestyle elements [4]. Three treatment options are available for lung cancer patients, including surgery, regional radiation, and integrated drug treatment. Surgical resection is the standard of care for patients with stage I and II, non-small-cell lung cancer (NSCLC), as well as select patients with stage IIIA [5]. Nevertheless, there are restricted drug therapies for patients with terminal diseases [6, 7]. Therefore, it is important to identify potential therapeutic targets that involved in the pathogenesis, progression, and migration of LC in order to improve treatment effects for LC patients.

The proteasome is a potential drug target for developing cancer treatment [8]. The ubiquitin (Ub)/proteasome system (UPS) is in charge of the protein (80–90%) degradation to keep effective cell function in eukaryotic cells [9]. Proteins special for degradation are primarily labeled with ubiquitin at one or more lysine surpluses [10, 11]. The identification and translocation of Polyubiquitinated proteins into the internal part of the proteasome 26S were made by the non-catalytic 19S subunits [12]. The Proteasome 26S subunit, non-ATPase 3 (PSMD3), is a component of the 26S proteasome, located at ch17q21 [13]. The expression of PSMD3 is found in many tissues [14] and takes part in numerous cellular processes [15].

Recently, PSMD3 was reported to participate in several human cancers by targeting various factors. PSMD3 controls breast cancer through the stabilization of HER2 from degradation [13]. PSMD3 exerts an oncogenic effect on chronic myeloid leukemia (CML) by stabilizing of nuclear factor-kappa B [16], therefore, PSMD3 is considered as a potential target for anti-cancer therapeutics in CML [17]. High degrees of PSMD3 are related to a worse overall survival (OS) in FLT3 mutated AML, indicating that PSMD3 may act as a prognostic biomarker in AML [18]. Moreover, the expression of PSMD3 was significantly higher in breast cancer tissue compared to normal tissues. The increased expression of PSMD3 was also related to poor prognosis for breast cancer patients [19]. The significant overexpression of PSMD3 was observed in Bladder cancer tissues when compared to normal bladder tissues [20]. These studies indicated that PSMD3 could be a hidden marker for cancer therapy or prognosis. Nevertheless, the effect of PSMD3 on LC progression is not yet clear.

The present study aims to research the role of PSMD3 in LC progression and explore the possible mechanisms involved in this regulation process, providing new targets for lung cancer therapy.

## Results

### PSMD3 is highly expressed in LC, and high PSMD3 predicts a poor prognosis

**Fig. 1:**
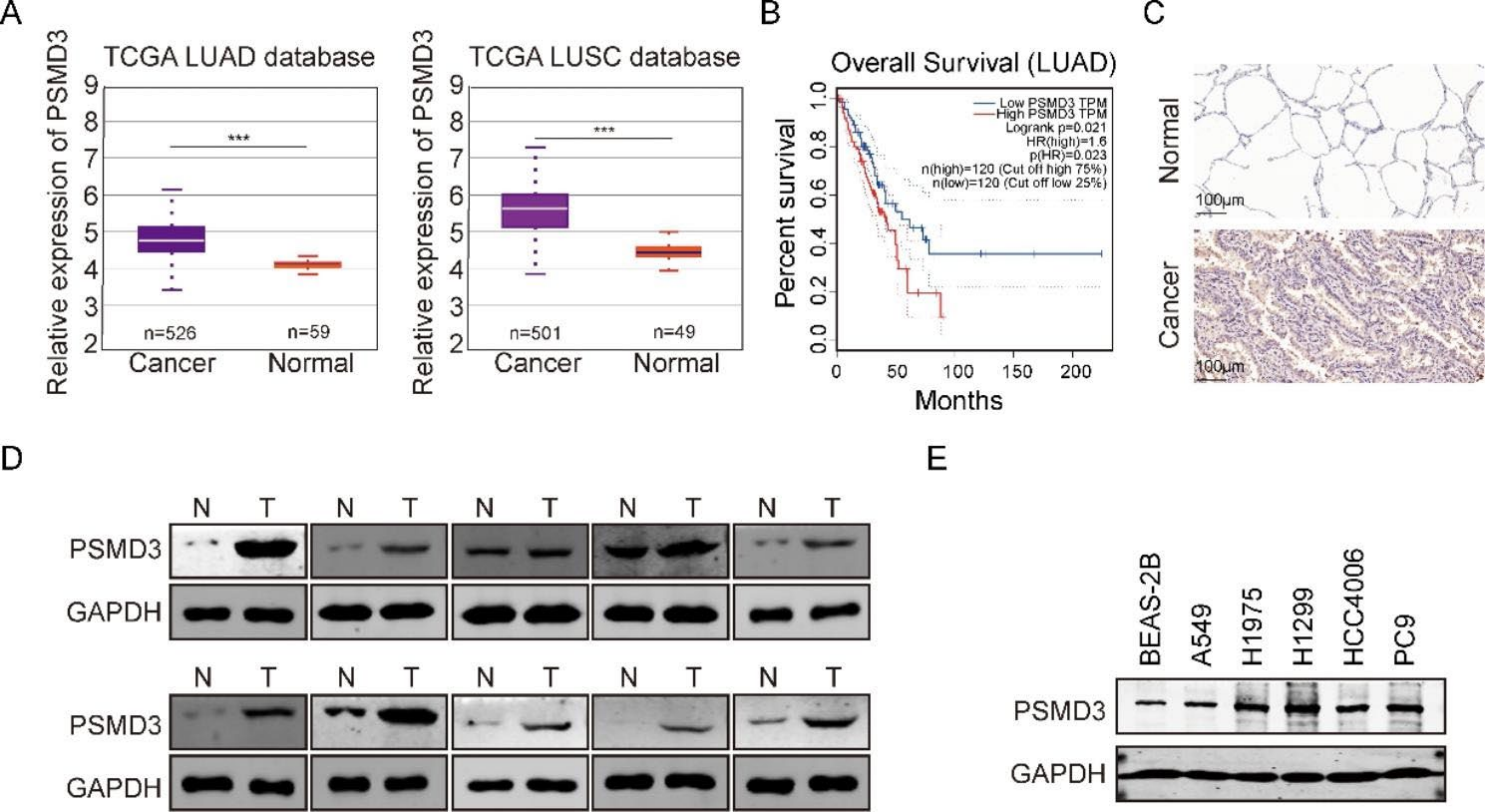
PSMD3 was upregulated in lung cancer and high PSMD3 indicates poor prognosis. (**A**) The expression of PSMD3 in LUAD and LUSC used samples from TCGA. (**B**) Kaplan-Meier analysis showed the low OS in high-PSMD3 TPM. (**C**) Representative images of IHC on PSMD3 expression in paired tumor and nearby noncancerous tissues from two typical patients. The scale bar stands for 100 μm. (**D**) The protein level of PSMD3 in 10 pairs normal versus LC tissue samples using western blot. GADPH served as internal control. N: Normal; T: tumor. (**E**) The protein level of PSMD3 in five LC cell lines and a normal lung cell. GADPH was internal control. * p < 0.05, ** p < 0.01, *** p < 0.001

To assess the transcription degree of PSMD3 in NSCLC tissues, we conducted analyses on the TCGA database. As shown in Fig. 1A, compared to control tissues, PSMD3 expression was significantly increased in LUAD and LUSC tissues. NSCLC patients with low levels of PSMD3 had longer OS than those with high levels (Fig. 1B). IHC results revealed that PSMD3 was obviously investigated in tumor tissues compared to normal ones (Fig. 1C). Western blot analysis conformed the upregulation of PSMD3 at the protein level in tumor tissues by using 10 pairs of normal lung tissues (N) and lung cancer tissues (T) (Fig. 1D). The clinical relevance of these findings was evaluated and showed a correlation between PSMD3 levels and pathologic T and N stage in LC patients (Supplementary Table 1). PSMD3 expression in five LC cell lines and normal lung cells was further tested using western blot. According to Fig. 1E, the high expression of PSMD3 was found in all LC cells when compared to BEAS-2B.

### PSMD3 drives cell proliferation in LC

**Fig. 2:**
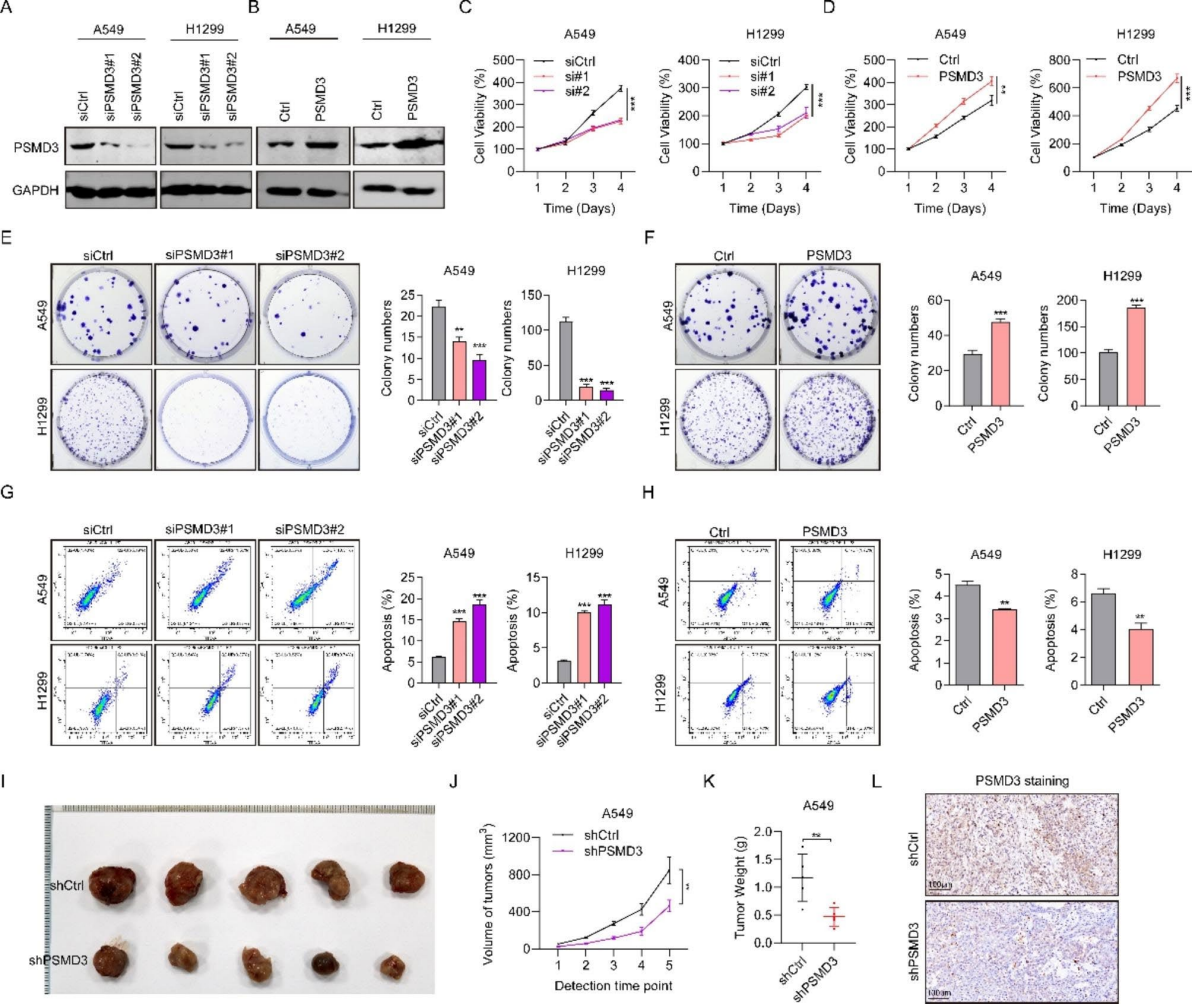
PSMD3 promotes cell proliferation in LC. (**A**-**B**) The protein levels of PSMD3 in two LC cell lines transfected with siPSMD3#1, siPSMD3#2, and siCtrl or pcDNA3.1-PSMD3, and Ctrl were determined using western blot. (**C**-**D**) Cell viability after PSMD3 knockdown of overexpression at 24, 48, 72 and 96 h were assessed by CCK-8 assay. (**E**-**F**) Cell colonies at 14 days in LC cells. (**G**-**H**) Cell apoptosis at 24 h after being treated with the suggested siRNAs and plasmids were assessed by flow cytometry. (**I**) Tumor size in nude mice between shCtrl and shPSMD3 groups were sacrificed at day 35. Images of the xenograft mice with tumors are displayed. (**J**) The measurement of tumor volumes of nude mice among three groups was made with digital calipers at day 7, 14, 21, 28, and 35. (**K**) The weight of xenograft tumors was measured among three mice groups at day 35. (**L**) Images of IHC staining of PSMD3 between two group tumor tissues. * p < 0.05, ** p < 0.01, *** p < 0.001

We knocked down the expression of PSMD3 with two siRNAs and elevated the expression of PSMD3 by using overexpressed plasmids in two LC cells, and the efficiency was confirmed with western blot (Fig. 2A-B). Cell propagation was measured using CCK-8 assay after siRNA or plasmids transfection. Inhibiting PSMD3 led to a significant decrease in cell viability in both cell lines, while ectopic expression of PSMD3 greatly increased cell viability (Fig. 2C-D). Consistently, PSMD3-knockdown cells generated greatly fewer colonies compared to controls, based on crystal staining, while PSMD3-overexpressed cells formed more colonies in both cell lines (Fig. 2E-F). Up-regulation of PSMD3 decreased cell apoptosis in H1299 and A549 cells, whereas down-regulation of PSMD3 resulted in an increase in apoptosis in both cell lines (Fig. 2G-H). To assess the role of PSMD3 in tumor development in vivo, we measured the tumor size of xenograft model mice with shCtrl and shPSMD3 at days 35 as shown in Fig. 2I. Vernier calipers were adopted to measure tumor volumes at days 7, 14, 21, 28, and 35. Our results displayed that the shPSMD3 group had a slower rate of tumor growth than shCtrl group (Fig. 2J). Furthermore, tumor weight in the shPSMD3 group was markedly lighter than the shCtrl group (Fig. 2K). Finally, we performed IHC staining in nude tumor tissues and found that PSMD3 staining was much weaker in the shPSMD3 group than in the shCtrl group (Fig. 2L). These outcomes indicated the oncogenic effect of PSMD3 on LC progression.

### PSMD3 drives cell migration and invasion in LC

**Fig. 3:**
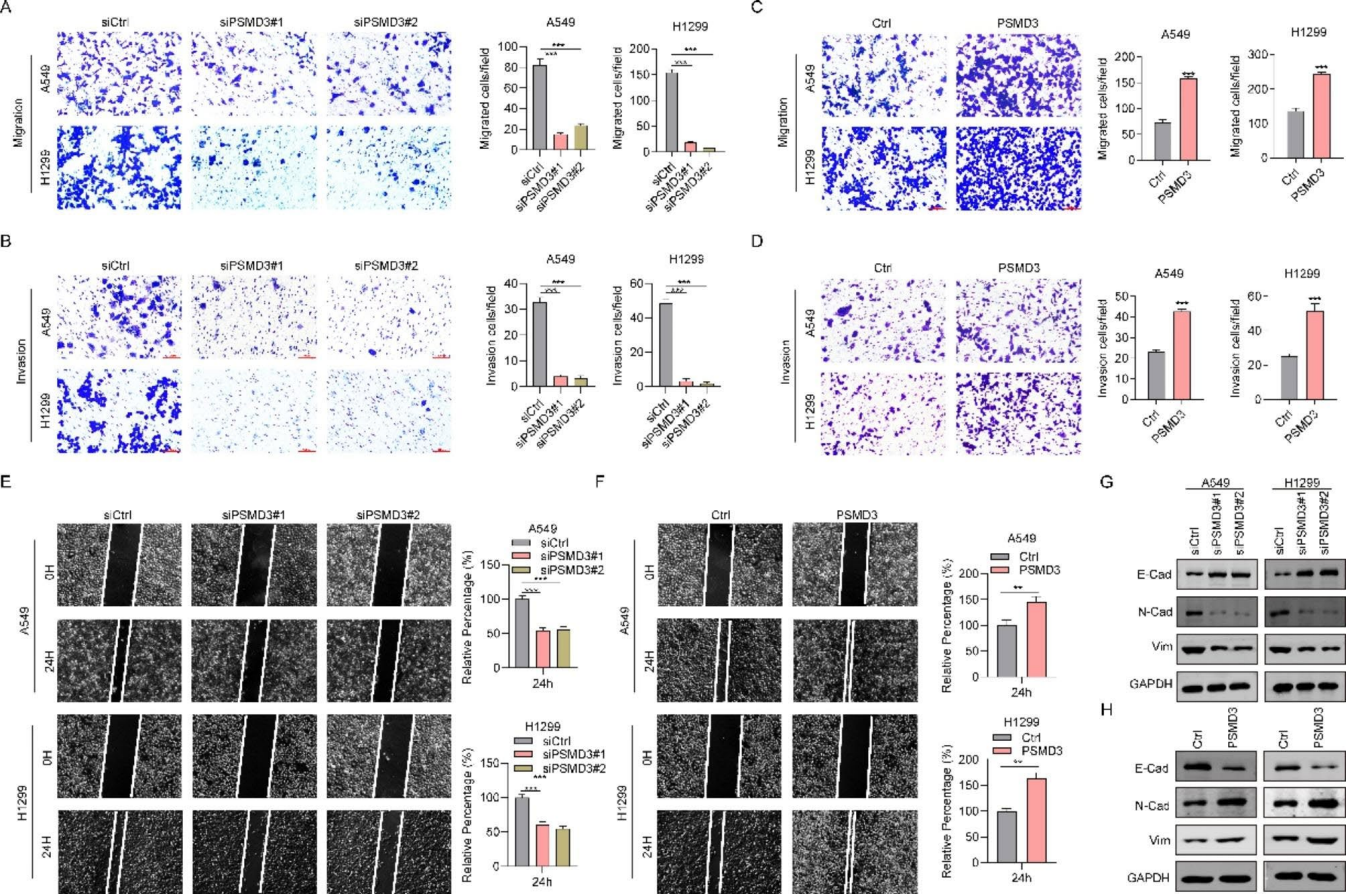
PSMD3 drives cell migration and invasion in LC. (**A**-**B**) Cell migration capacity was detected using Transwell assay. (**C**-**D**) Cell invasion capacity was determined using Transwell assay. (**E**-**F**) Cell migration ability was measured using Wound healing assay. (**G-H**) The protein level of EMT markers (N-cadherin, VIMENTIN and E-cadherin) in A549 and H1299 cell lines with PSMD3 knockdown or PSMD3 overexpression, which were determined by western blot. GADPH was internal control. * p < 0.05, ** p < 0.01, *** p < 0.001

The Transwell assays demonstrated that reducing PSMD3 levels decreased cell migration and invasion in two LC cell lines (Fig. 3A-B), whereas PSMD3 overexpression had the opposite effect (Fig. 3C-D). Wound-healing experiments also revealed that cells with PSMD3 knockdown healed the wounds slower than shCtrl cells (Fig. 3E), whereas PSMD3 overexpressed cells healed the region of the wound greatly faster than the Ctrl cells (Fig. 3F). These findings suggested that PSMD3 drives cell migration and invasion in LC. Furthermore, we exmamined the levels of several EMT markers in A549 and H1299 cells. Intriguingly, knocking down PSMD3 reduced the expression of N-cadherin and VIMENTIN, while stimulating E-cadherin expression. Conversely, overexpression of PSMD3 markedly promoted the expression of N-cadherin and VIMENTIN but inhibited E-cadherin expression (Fig. 3G-H).

### PSMD3 interacts with ILF3

**Fig. 4:**
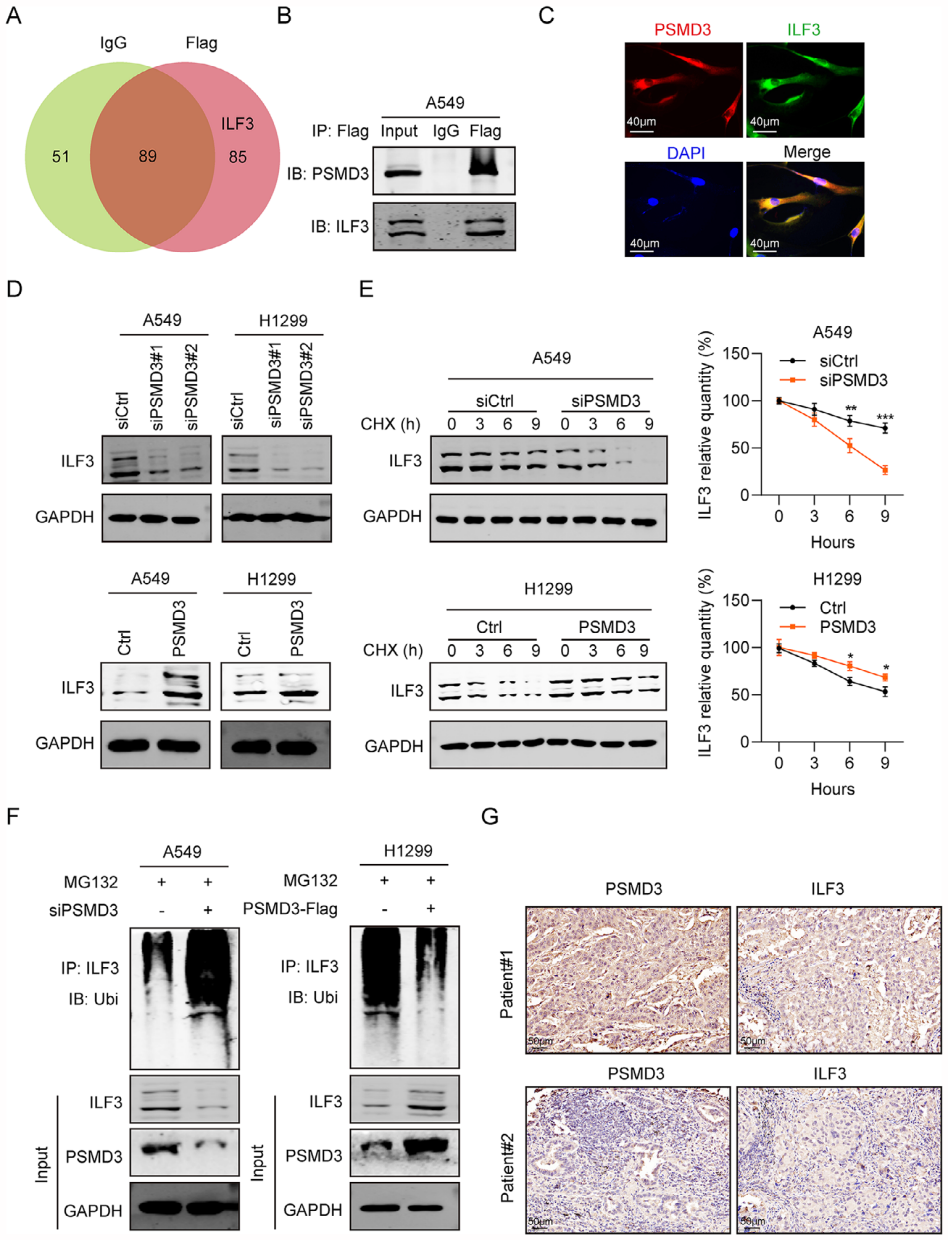
PSMD3 binds to ILF3. (**A**) Venn diagram of common genes using RNA pull-down assay and mass spectrometry for identification of PSMD3-associated proteins with A549 cell lysates. (**B**) Co-IP was made with the ILF3 antibody in LC cells lentivirally transduced with the empty vector or vector encoding PSMD3-flag. WB was adopted to analyze immunoprecipitants. (**C**) Immunofluorescence staining for PSMD3-flag (red) and ILF3 (green) in LC cells. (**D**) The protein levels of ILF3 in LC cells with PSMD3 knockdown or overexpression. (**E**) Reduction of ILF3 protein stability in A549 cells with PSMD3 knockdown and in H1299 cells with PSMD3 overexpression. (**F**) The poly-ubiquitination of ILF3 in PSMD3 overexpression or knockdown cells were assessed using the ubiquitination assay. (**G**) The expression of PSMD3 and ILF3 in LC clinical tissues were analyzed using IHC staining. * p < 0.05, ** p < 0.01, *** p < 0.001

We conducted co-immunoprecipitation and mass spectrometry assays to identify proteins that interact with PSMD3. We identified 225 proteins, among which ILF3 was found to be highly ranked (Fig. 4A). ILF3 is an RNA-binding protein known for its involvement in innate immunity by participating in cellular antiviral responses [21]. Our study found that ILF3 co-precipitated with PSMD3-flag protein, and vice versa, in two LC cell lines (Fig. 4B). Immunofluorescence analyses confirmed the relationship between PSMD3 and ILF3, and confocal microscopy showed that PSMD3 and ILF3 were co-localized within cytoplasm (Fig. 4C). Furthermore, we observed that the level of ILF3 protein was significantly decreased after PSMD3 knockdown but increased greatly following PSMD3 overexpression (Fig. 4D). To investigate whether PSMD3 controls the stability of ILF3 protein, LC cells were treated with protein synthesis inhibitor Cycloheximide and acquired proteins at 0 h, 3 h, 6 h, and 9 h. It was observed that ILF3 protein degraded faster in the siPSMD3 groups than the siCtrl groups (Fig. 4E), indicating that depletion of PSMD3 decreased the stability of ILF3 protein.

An endogenous ubiquitination assay showed that overexpression of PSMD3 facilitated ubiquitination of endogenous ILF3, but knockdown of PSMD3 promoted deubiquitination of endogenous ILF3 (Fig. 4F). Additionally, our analyses revealed high expression of both PSMD3 and ILF3 among clinical patients with lung cancer (Fig. 4G). In conclusion, these findings suggested the interaction between PSMD3 and ILF3 in lung cancer cells.

### PSMD3 drives cell proliferation and migration through ILF3

**Fig. 5:**
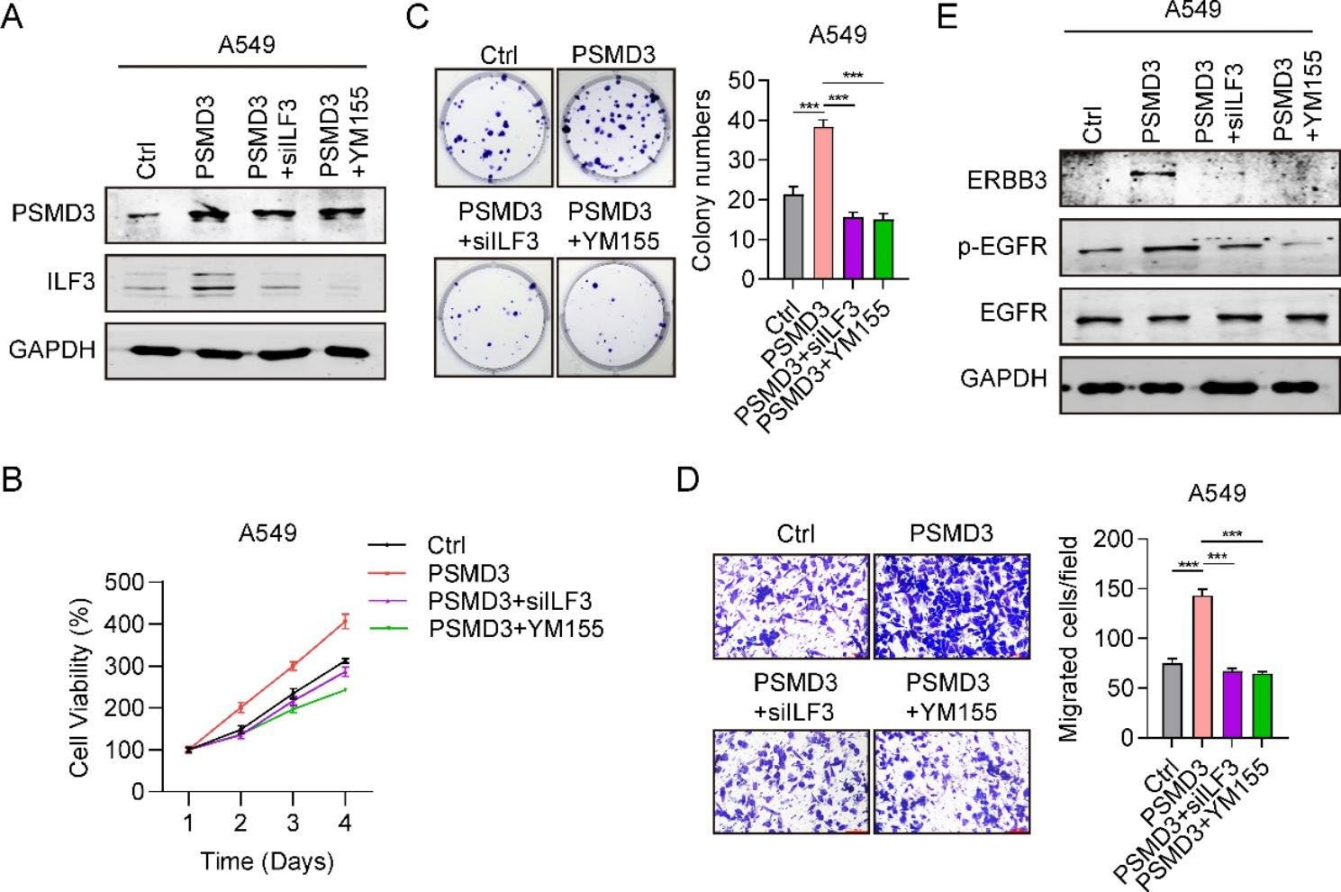
PSMD3 promotes cell proliferation and migration by ILF3. (**A**) The protein level of PSMD3 and ILF-3 in LC cells treated with Ctrl, pcDNA3.1-PSMD3, pcDNA3.1-PSMD3 + siILF3 and pcDNA3.1-PSMD3 + YM155. (**B**) Cell viability in four groups. (**C**) Cell colony numbers in four groups. (**D**) Migrated cells in four groups using Transwell assay. (**E**) The protein levels of ERBB3, pEGFR and EGFR in A549 cells among four groups. * p < 0.05, ** p < 0.01, *** p < 0.001

SiRNAs targeting ILF3 (siILF3#1) and ILF3 inhibitor (YM155) were all used to knock down ILF3 in H1299 and A549 cells. PSMD3 and ILF3 protein levels were greatly increased in PSMD3 overexpressed cells compared to Ctrl cells. However, compared to PSMD3 overexpression cells, the protein levels of PSMD3 and ILF3 were significantly decreased in PSMD3 + siILF3 and PSMD3 + YM155 groups (Fig. 5A). The CCK-8 test displayed that PSMD3 overexpression promoted cell proliferation in H1299 and A540 cells, and siILF3 and YM155 inhibited cell proliferation to Ctrl levels (Fig. 5B). The results of the colony formation assay suggested that overexpression of PSMD3 increased the ability of H1299 and A549 cells to form colonies, while siILF3 and YM155 inhibited this process (Fig. 5C). Consistently, cell migration ability was also reduced by siILF3 and YM155 (Fig. 5D). Previous studies have reported that ILF3 promoted lung cancer by activating the EGFR-mediated cellular pathway [22]. Therefore, we assessed the proteins related to EGFR. Western blot analysis revealed a significant increase in ERBB3 (HER3) and p-EGFR expression after PSMD3 overexpression, whereas siILF3 and YM155 suppressed their expression (Fig. 5E). The expression of EGFR did not show any significant changes among these groups. These outcomes indicated that PSMD3 drived cell proliferation and migration through ILF3.

### PSMD3 promotes tumor growth in vivo through ILF3

**Fig. 6:**
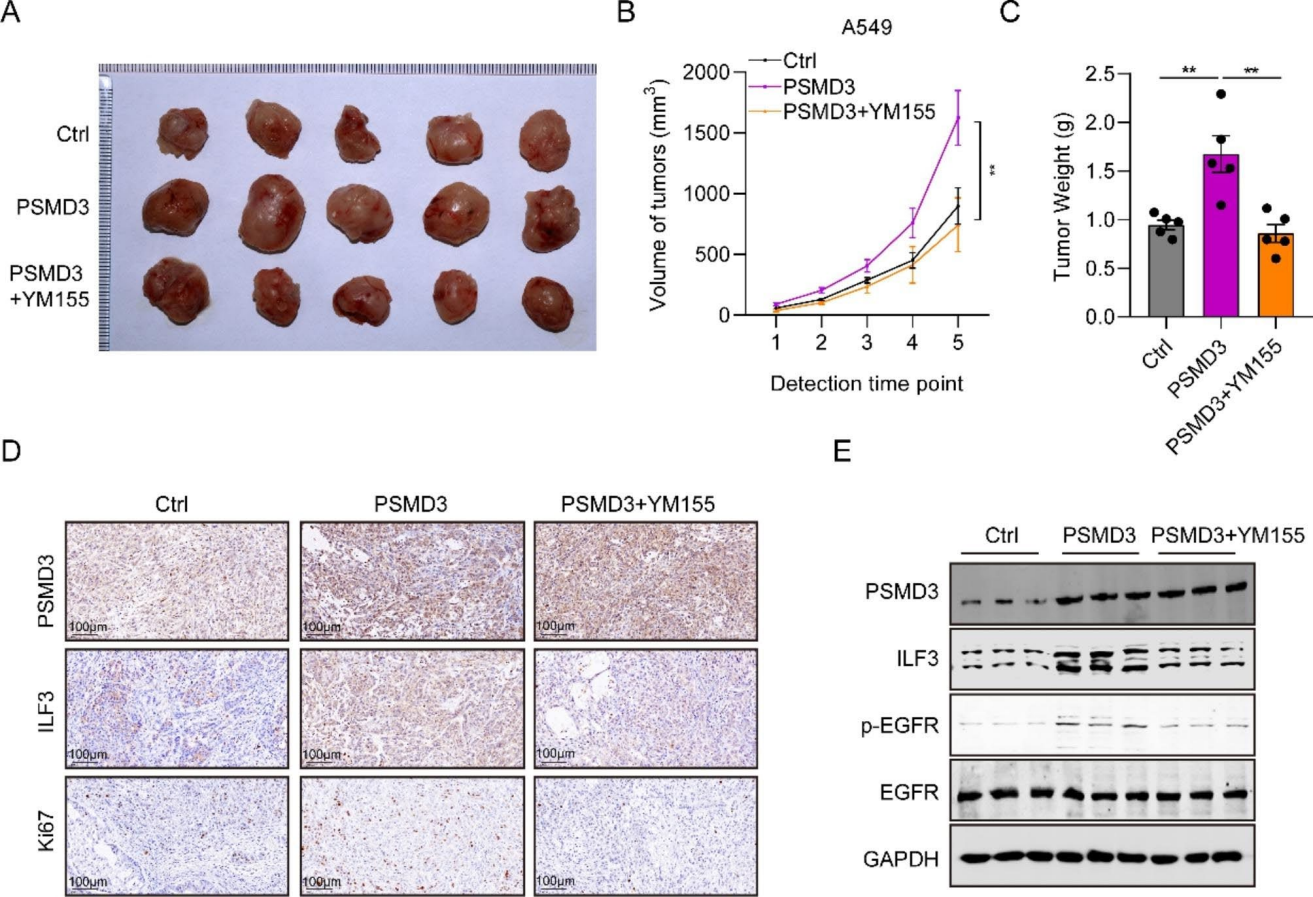
PSMD3 drives tumor growth in vivo by ILF3. (**A**) Tumor size in nude mice among Ctrl, PSMD3, and PSMD3 + YM155 groups was sacrificed at day 42. Images of the xenograft mice with tumors are displayed. (**B**) The measurement of tumor volumes of nude mice among three groups was made with digital calipers at day 7, 14, 21, 28, 35 and 42. (**C**) The weight of xenograft tumors was measured among three mice groups at day 42. (**D**) IHC images of ki67, PSMD3 and ILF3 in three group tumor tissues. Scale bars, 100 μm. (**E**) The protein expression of PSMD3, ILF3, pEGFR and EGFR in tumor tissues among three groups were detected using western blot. ** p < 0.01

To assess the role of PSMD3 in tumor development in vivo, Ctrl, pcDNA3.1-PSMD3 overexpression plasmids (PSMD3 groups) or/and ILF3 inhibitor YM155 (PSMD3 + YM155), transfected A549 cells were used in tumor xenograft model mice. Tumor size was measured using Vernier calipers at day 7, 14, 21, 28 and 35 as shown in Fig. 6A. The results indicated that PSMD3 greatly increased tumor size while YM155 significantly decreased it. Our outcomes displayed that the tumor growth rate in the PSMD3 group was greatly faster than Ctrl, whereas that in the PSMD3 + YM155 group was slower compared to the PSMD3 group (Fig. 6B). Additionally, we observed consistent results for the trend of tumor weight (Fig. 6C). We also performed IHC staining in nude tumor tissues and found PSMD3 and ILF3 staining were much stronger in the PSMD3 group than Ctrl group, while these in the PSMD3 + YM155 group were slighter than PSMD3 group (Fig. 6D). The protein levels of PSMD3, ILF3 and pEGFR significantly grew in tumor tissues of the PSMD3 group compared to Ctrl group, which greatly decreased in PSMD3 + YM155 group compared to PSMD3 group (Fig. 6E). These results suggested that PSMD3 promoted tumor growth through ILF3 in vivo.

## Discussion

The ubiquitin (Ub)/proteasome system (UPS) exerts crucial effects on regulating various biological processes, including cell cycle processes, cell development, and migration, except for regulating DNA destruction, transcription, and apoptosis [23, 24]. The 26S proteasome is composed of a 20S core and a 19S regulator that includes a base with six ATPase subunits and two non-ATPase subunits along with a lid consisting of up to ten non-ATPase subunits. One of the components of the lid is PSMD3 (Rpn3) [25–27].

Numerous studies showed that each of the 26S proteasome subunits was involved in lung cancer progression. PSMD1 drove the viability and repressed the apoptosis of LUAD cells through the stabilization of PINK1 [28]. Suppressing PSMD2 reduced proteasome activity and induced growth inhibition control and apoptosis in LC cells [29]. Similarly, knocking down PSMD7 expression significantly reduced both cell proliferation and tumor development in vitro and in vivo by controlling the p53 pathway [30]. In addition, knockdown of PSMD14 led to increased levels of cleaved caspase-3 and subsequent induction of cell apoptosis in H1299 cells [31]. Highly expressed PSMD2 and SMD14 were greatly related to lymph node metastases and TNM phase of lung adenocarcinoma [32, 33], while high expression of PSMD2, PSMD7, PSMD14 were linked to poor OS and/ or disease-free survival (DFS) among these patients [30, 32, 33].

This study observes the protein expression of PSMD3 markedly grew in lung cancer samples from TCGA database and clinical patients. And high expression of PSMD3 indicates poor overall survival of LC patients. The upregulation of PSMD3 expression was found in five different LC cell lines comparing to normal lung cells. Knockdown of PSMD3 using siRNAs in H1299 and A549 cells decreased cell proliferation and invasion capacity, whereas the apoptosis rate was promoted. In the contrast, PSMD3 overexpression got the opposite results. Knockdown of PSMD3 also inhibited tumor growth in vivo.

Furthermore, we utilized Co-IP and MS assays to investigate the potential protein interactions of PSMD3. Interleukin enhancer-binding factor 3 (ILF3), a member of the DRBPs family, caused our interest due to its effect on cancer progression [34, 35], and it is crucial in RNA metabolism, including maintaining the stability of mRNA [36]. ILF3 overexpression could control gastric cancer progression via PI3K/AKT/mTOR pathway [35]. ILF3-mediated LLPS is a physiological roles in tumor microenvironment remodeling, and it is a potential target for esophageal cancer [37]. Additional, ILF3 overexpression was related to prediction of survival in non-small cell lung cancer [38].

Our study confirmed the interaction between PSMD3 and ILF3 using co-immunoprecipitation assays and confocal microscopy. ILF3 expression was greatly reduced in LC cells after the knockdown of PSMD3 and increased following PSMD3 overexpression using RT-qPCR and western blot. PSMD3 also regulated ILF3 protein stability and facilitated the ubiquitination of endogenous ILF3. The expression of PSMD3 and ILF3 were all upregulated in LC clinical samples.

To further confirm the role of ILF3 on PSMD3-promoted LC progression, we introduced ILF3 inhibitor in vitro and in vivo assay. Both siRNAs targeting ILF3 (siILF3) and ILF3 inhibitor YM155 could suppress promoting effect of PSMD3 in LC cell proliferation and migration. Tumor xenograft model assay also got similar results. PSMD3 overexpression significantly promoted tumor growth, while YM155 considerably slowed tumor growth. However, we were unable to determine LC progression mediated by ILF3 in mice models as we did not have access to ILF3 knockdown mice, which is a limitation of our study.

Cheng et al. disclosed that the Epidermal growth factor receptor (EGFR) could induce ILF3 expression in LC cells, and the inhibitor of ILF3, YM155, reduced EGFR expression [22]. Hence, after detecting the genes pression of related to EGFR-mediated cellular pathway, western blot outcomes displayed that ERRB3 and p-EGFR were all upregulated following PSMD3 overexpression but down regulated following siILF3 and TM155 treatment.

## Conclusions

In summary, we found PSMD3 was significantly upregulated in LC cells and high PSMD3 expression indicates poor prognosis in LC. Furthermore, PSMD3 is closely related to the progression of LC and could promotes the cell proliferation, migration, and invasion of LC cells. In addition, PSMD3 can regulate ILF3 protein stability and facilitate the ubiquitination of endogenous ILF3 in LC, which exerted an oncogenic effect on tumor growth and proliferation. Therefore, targeting the PSMD3/ILF3 pathway may provide novel strategies for diagnosing and treating LC.

## Methods

### Samples from the TCGA database and clinical tissues

The retrieval and assessment of the mRNA expression of PSMD3 from TCGA databases were made for 59 normal lung tissues and 526 lung adenocarcinoma tissues (LUAD), as well as 49 normal lung tissues, and 501 lung squamous cell carcinoma (LUSC) tissues. Based on the average level of PSMD3, the samples were classified into PSMD3 low (group I) and PSMD3 high (group II) groups.

Ten pairs of NSCLC specimens and adjacent noncancerous tissues were collected from patients who underwent curative operation at the Department of Thoracic Surgery, China-Japan Friendship Hospital (Beijing, China) between January 2021 and December 2021. Tumor samples were carefully isolated during the operation and immediately frozen and stored at -80 °C for further analysis. All NSCLC patients in the research provided written permission. The Department of Thoracic Surgery, China-Japan Friendship Hospital (Beijing, China) Institutional Review Board (No. 2019-95-K63-1) approved the experimental steps.

### Cell culture

The culture of human LUAD cells H1299 and A549 was made in DMEM medium supplemented by FBS (10%), penicillin (100 U/mL), and streptomycin (100 µg/mL).

### Cell transfection

The transfection of H1299 and A549 cells was made with siRNAs (100nM) targeting PSMD3 or ILF13 with Lipofectamine 2000 (Invitrogen, USA) based on the producer’s guidance. The siRNA sequences were shown below: siPSMD3#1: 5’-CCAUGAGGUUUCCUCCCAAAU-3’; siPSMD3#2: 5’-GCAGGGCUUCUUCACUUCAAA3’; siILF3: 5’-UUGAUGGACAGAAGUUCCAAG-3’; and siCtrl: 5’-UUCUCCGAACGUGUCACGU-3’. For PSMD3 overexpression, the full-length CDS of the PSMD3 gene (1605 bp) was inserted into the pcDNA3.1 vector to produce the pcDNA3.1 + PSMD3 overexpression vector. A pipette-type incorporator MP-100 was adopted to transfect the overexpression vectors (2 μg) into H1299 and A549 cells.

### Immunohistochemistry (IHC)

The overnight incubation of tissue sections from clinical patients or xenografts was made with anti-PSMD3 antibody (Affinity biosciences, DF3645, 1:100), anti-ILF3 (Abcam, ab92355, 1:100), and anti-Ki67 at 4 °C. 0.3% H_2_O_2_ was used for blocking endogenous peroxidases for 15 min. After three times of section washing, streptavidin and biotin-conjugated HRP was put into the tissues for incubation for 1 h at 25℃ and then stained with 3’-diaminobenzidine for 30 min and stopped by 5-minute rinsing in H_2_O. IHC staining results were imaged with a Leica upright microscope.

Two experienced pathologists conducted an independent assessment of immunohistochemical outcomes, which were then scored based on Friedrich’s standard. The ratio of positive tumor cells was assessed with a mark of 0 (0–5%), 1 (6–20%), 2 (21–50%), or 3 (51–100%). The marks of the staining intensity and the ratio of positive cells were multiplied to obtain the final score. A final score ≤ 1 indicated low expression, while a score > 1 was defined as a high expression.

### Western blot

The extraction of overall proteins of tissues and cells was made with FLAG lysis buffer with a protease inhibitor cocktail. A BCA kit was used to measure protein concentrations. Proteins were electrophoresis, transferred, and blocked, then incubated with the diluted primary antibodies at 4 °C overnight. PSMD3 (Affinity biosciences, DF3645, 1:1000), ILF3 (Abcam, ab92355, 1:1000), ERBB3 (Affinity biosciences, DF6144, 1:1000), p-EGFR (Abcam, ab32430, 1:1000), EGFR (Abcam, ab32077, 1:1000), and GAPDH (Cell signaling, 21185, 1:1000) were all used in this study. Horseradish peroxidase-labeled secondary antibodies was adopted to incubate the membranes after extensive washes, and a strengthened chemiluminescent kit with a ChemiDoc XRS testing system was adopted to test signals. ImageJ software was adopted to analyze the signals.

### CCK-8 assay

The measurement of cell viability was made with CCK-8 assay. In detail, the seeding of LC cells (2 × 10^4^ cells per well) was made in 24-well culture plates after being transfected with suggested siRNAs, plasmids, or inhibitors.

### Cell colony formation assay

After the treatment of LC cells with the suggested siRNAs, plasmids, or inhibitor, the seeding of cells (1000 cells per well) into 12-well plates was made in triplicate, followed by 14-day culture until the cells grew in colonies. After fixing the cells for 10 minutes, they were stained with crystal violet and the colonies were counted using Image J software.

### Transwell assay

To evaluate cell migration and invasion, a Transwell chamber was used with or without 10 µg/mL Matrigel. At 48-hour transfection, cells developed in the upper chamber. Medium with 10% FBS was filled in the lower chamber. After 48 h, the migrating or invading cells were fixed with methanol, stained with 0.1% crystal violet, and counted under a microscope.

### Wound healing assay

LC cells were transfected with siRNAs or plasmids and then cultured for 24 h. The aspiration of the medium was made, and a 20-µl pipette tip was adopted to scrap the cell-coated surface. The wounded surface was washed twice with culture medium warmed to 37 °C, and then incubated in an incubator microscope at 37 °C with 5% CO_2 _for 24 h to allow for wound healing. The movement of cells to wounded regions was photographed at 24 h. A computer-assisted image analyzer was used to analyze the pictures. The migration rate was calculated as follows: % of scratch closure = a-b/a, where (a) represents the region from margins to wound, and (b) represents the cell-free region.

### Flow cytometry for cell apoptosis

The 24-hour transfection of cells was made with suggested siRNAs or plasmids, followed by collection in cell binding buffer (100 µL). Next, the cells were stained using PE Annexin V or 7AAD from Annexin V Apoptosis Detection Kit and analyzed by Flow cytometry (Beckman Coulter).

### Co-immunoprecipitation (CO-IP) and mass spectrometry (MS)

The lysis of overnight cultivated cells was made in a Co-IP buffer. Protein A of 10 µL or G agarose beads was added for 1 h at 4 °C to preclear cell lysates. IgG was adopted as a negative regulation. Next, another 2-hour incubation of 15 µL protein A or beads were made at 4 °C. After centrifugation at 1200× g, cell lysis buffer was used to collect the wash the pellet twice. Resolved on 12% SDS-PAGE, immunoprecipitated proteins were discussed by Western blot. Differential bands were identified using Mass Spectrometry (MS).

### Immunofluorescence (IF) staining

The 10-minute fixture of cells was made with 4% paraformaldehyde at 25 ℃, followed by 5-minute permeabilization with 0.1% Triton X-100. Bovine serum albumin (BSA) of 2% was used to block samples for 30 min. After diluting 1/100 with 1% BSA in PBS, the overnight incubation of primary antibodies was made at 4 °C. After washing, the cells were incubated for an additional  3-minute before staining the nuclei with DAPI. Finally, images were acquired using a confocal laser microscope.

### CHX protein stability

LC cells were treated with indicated siRNAs or plasmids and 1 µg/mL Cycloheximide (CHX). The proteins were separated at 0 h, 3 h, 6 h, 9 h, and western blot was adopted for detecting ILF3 expression, and the intensity was quantified by Image J software [22].

### Ubiquitination assay

The 24-hour co-transfection of ILF3-Flag, HA-UB plasmids with or without PSMD3 was made in HEK293T cells. After a subsequent 4-hour incubation with 10 µM MG132, the collection and lysis of cells were made. The ubiquitination status of ILF3 was analyzed with western blotting.

### Animal experiments

In the present study, 25 male BALB/c nude mice were provided by Beijing Vital River Laboratory Animal Technology Co., Ltd, which was approved by the Animal Ethics Committee of China-Japan Friendship Hospital. One side, A549 cells transfected with shCtrl and shPSMD3 were harvested at 24 h after transfection. The suspension of 2 × 10^6^ transfected tumor cells in 50 µL PBS were subcutaneous injection to the flanks of mouse (n = 5). Vernier calipers were adopted to measure the tumor growth curves at days 7, 14, 21, 28, and 35. On the other side, A549 cells transfected with Ctrl, pcDNA3.1-PSMD3 and pcDNA3.1-PSMD3 + YM155 were harvested at 24 h after transfection. The suspension of 2 × 10^6^ transfected tumor cells in 50 µL PBS and subcutaneous injection of 50 µL Matrigel matrix into both sides of the flanks of every mouse were found. The mice were devided into three groups randomly with 5 mice per group. Tumor growth curves were measured using Vernier calipers on days 7, 14, 21, 28, and 35. Gene expression in these tumor tissues were analyzed using IHC and western blot.

### Statistical analysis

SPSS version 18.0 (SPSS, Inc., Chicago, IL, USA) was adopted to analyze the data. The expression differences between lung adenocarcinoma tissues and nearby normal tissues were explored by proposing the Mann-Whitney U test. Calculated by the Kaplan-Meier approach, the OS and DFS distribution were analyzed by the log-rank test.

## Electronic supplementary material

Below is the link to the electronic supplementary material.

Supplementary Material 1

## Data Availability

The datasets generated and analyzed in this research are available from the corresponding author on reasonable request.

## Abbreviations

LC: lung cancer
NSCLC: non-small-cell lung cancer
LUAD: lung adenocarcinoma tissues
LUSC: lung squamous cell carcinoma
PS: ubiquitin (Ub)/proteasome system
PSMD3: Proteasome 26S subunit, non-ATPase 3
ILF3: Interleukin enhancer-binding factor 3
EGFR: Epidermal growth factor receptor
IHC: Immunohistochemistry
CO-IP: Co-Immunoprecipitation
MS: mass spectrometry
IF staining: Immunofluorescence staining
CHX: Cycloheximide
