# Supplementary material for: PSMD3-ILF3 signaling cascade drives lung cancer cell proliferation and migration
Source: Biol Direct. 2023 Jun 19;18:33. doi: 10.1186/s13062-023-00389-3 (PMC10278297; doi:10.1186/s13062-023-00389-3)
Supplement: Supplementary file 1 — Supplementary Material 1 [file 13062_2023_389_MOESM1_ESM.docx]

Supplementary Table 1. Correlation between PSMD3 expression and clinic pathological features in lung cancers based on TCGA database.

| **Characteristics** | **Low expression of PSMD3** | **High expression of PSMD3** | **P value** |
| --- | --- | --- | --- |
| n | 520 | 521 |  |
| Age, n (%) |  |  | 0.173 |
| <= 65 | 213 (21%) | 235 (23.2%) |  |
| > 65 | 293 (28.9%) | 272 (26.9%) |  |
| Gender, n (%) |  |  | < 0.001 |
| Female | 240 (23.1%) | 180 (17.3%) |  |
| Male | 280 (26.9%) | 341 (32.8%) |  |
| Pathologic stage, n (%) |  |  | 0.053 |
| Stage I | 290 (28.2%) | 251 (24.4%) |  |
| Stage II | 128 (12.4%) | 159 (15.5%) |  |
| Stage III | 76 (7.4%) | 92 (8.9%) |  |
| Stage IV | 17 (1.7%) | 16 (1.6%) |  |
| Pathologic T stage, n (%) |  |  | 0.043 |
| T1 | 164 (15.8%) | 126 (12.1%) |  |
| T2 | 278 (26.8%) | 308 (29.7%) |  |
| T3 | 58 (5.6%) | 62 (6%) |  |
| T4 | 17 (1.6%) | 25 (2.4%) |  |
| Pathologic N stage, n (%) |  |  | 0.021 |
| N0 | 347 (34.1%) | 323 (31.7%) |  |
| N1 | 93 (9.1%) | 135 (13.2%) |  |
| N2 | 59 (5.8%) | 55 (5.4%) |  |
| N3 | 2 (0.2%) | 5 (0.5%) |  |
| Pathologic M stage, n (%) |  |  | 0.781 |
| M0 | 369 (45.6%) | 408 (50.4%) |  |
| M1 | 16 (2%) | 16 (2%) |  |
| Smoker, n (%) |  |  | 0.040 |
| No | 57 (5.6%) | 38 (3.7%) |  |
| Yes | 450 (44.3%) | 470 (46.3%) |  |
